# Young People’s Experiences of Engaging With Fitspiration on Instagram: Gendered Perspective

**DOI:** 10.2196/17811

**Published:** 2021-10-04

**Authors:** Joanne Mayoh, Ian Jones

**Affiliations:** 1 Department of Sport and Event Management Bournemouth University Poole United Kingdom

**Keywords:** social media, gender, physical fitness, women’s health, men’s health, body ideals

## Abstract

**Background:**

Fitness inspiration or *fitspiration* is a term used to describe web-based images of fit people, people in the gym, health foods, or inspirational quotes relating to diet and fitness being shared and consumed via visual social media. The popularity of this content is most notable via the *Instagram* platform. Currently, the majority of fitspiration research has focused on women’s experiences; however, increasingly, studies have pointed to the need to explore the gendered ways by which people engage with this content.

**Objective:**

The aim of this study is to explore how young men and women engage in fitspiration content on Instagram and provide a gendered analysis of how and why they consume this content.

**Methods:**

This study used a cross-sectional web-based survey (N=1213) of UK-based fitspiration users aged 18-24 years consisting of closed-ended questions to capture quantitative data.

**Results:**

The majority actively using Instagram for fitspiration (therefore eligible participants) were women (826/1175, 70.30%). Men were more likely to view content posted by athletes (χ^2^_1, N=1153_=71.8; *P*=.001) and bodybuilders (χ^2^_1, N=1153_=32.8; *P*<.001), whereas women were more likely to view content related to weight loss (χ^2^_1, N=1153_=36.8; *P*<.001), diet plans (χ^2^_1, N=1153_=11.9; *P*<.001), and celebrities’ content (χ^2^_1, N=1153_=33.5; *P*<.001). Men were more likely to use fitspiration as a source of inspiration to exercise to gain muscle or get stronger (χ^2^_1, N=1147_=17.9; *P*<.001), whereas women were more likely to use fitspiration as inspiration for healthy eating (χ^2^_1, N=1147_=37.7; *P*<.001), or to exercise to diet or lose weight (χ^2^_1, N=1147_=13.5; *P*<.001). Women were more likely to engage in passive behaviors such as viewing content on their feed (χ^2^_1, N=1139_=7.9; *P*=.005) or scrolling through accounts (χ^2^_1, N=1139_=15.2; *P*<.001), whereas men were more likely to engage in active consumption by tagging fitspiration accounts in posts (χ^2^_1, N=1139_=7.2; *P*=.007), commenting on posts (χ^2^_1, N=1139_=8.1; *P*=.004), and posting fitspiration content (χ^2^_1, N=1139_=6.4; *P*=.01).

**Conclusions:**

Female fitspiration consumers engaged with content that reinforced the feminine *thin but shapely* ideal, whereas male users sought out content that reinforced the masculine *muscular* ideal. Male users were more likely to engage actively with content (eg, posting fitspiration content), while female users were more likely to engage passively (eg, scrolling through accounts, posts, or images). Future research should consider how fitspiration consumption reflects and reproduces oppressive gender ideology.

## Introduction

### Background

Social media is becoming embedded within our everyday lives, with 68% of people and 98% of those aged 16-24 years within the United Kingdom using web-based social networking platforms [1]. Among their many purposes, web-based social media platforms are used for communication, community building, information gathering, and as a source of inspiration within multiple contexts. One such context is that of *fitspiration* or *fitspo*, commonly used abbreviations for the term *fitness inspiration* [2], which describes web-based content such as images of fit people, people in the gym, health foods, or inspirational quotes relating to diet and fitness shared and consumed habitually via social media. Increasingly, academic research has demonstrated numerous perceived positive outcomes associated with consuming the fitspiration content on social media [2-5]. These studies explore how web-based fitspiration can provide wider access to reliable health information [2-5] and motivate individuals to engage in healthy behaviors [3], including by providing motivation to exercise [4] and in helping to create a sense of community [2].

While fitspiration is widespread on multitudinous social media platforms, it is particularly common on Instagram [6]. Instagram is a predominantly mobile app, with over 400 million global users [7] including 29% of women and 26% of men in the United Kingdom [8]. With regard to fitspiration, a recent Instagram search (July 11, 2019) of #fitspo yielded over 66 million posts [9] demonstrating the popularity of this specific content. A key reason for complementarity between this visual platform and fitspiration is that Instagram has a unique focus on sharing and consuming *snapshot photography* or self-selected images in which individuals are portrayed in an attractive light [10] making it particularly harmonious with imagery that showcases fit and healthy *ideal* bodies and lives, reinforcing social norms regarding health and fitness. Furthermore, Instagram offers users the opportunity to edit and apply filters to images to distort reality into an ideal to be shared, making the potential impact on well-being and appearance concerns more pronounced when compared with other platforms [11]. It is therefore imperative that research focuses on this unique platform to adequately inform policy regarding the health and well-being of young adults (<24 years).

Users of Instagram are exposed to multiple *idealized* images of peers and other people or organizations (with public accounts) actively shaping visual ideas about beauty regarding *acceptable* or *desirable* bodies [12]. Those who engage with fitspiration on Instagram specifically (eg, by following or scrolling through specific accounts or searching for hashtags) are exposed to idealized *fit* bodies and unsubstantiated, often unrealistic (and occasionally incorrect) advice regarding diet and fitness, shaping the body’s ideal narrative and reinforcing social norms regarding the body. Research exploring the nature of fitspiration on social media and Instagram has demonstrated that the content displays multiple potentially harmful themes and includes objectifying images that depict idealized bodies [2-4], can depict extreme and often restrictive healthy-eating content linked to disordered eating [13], and can promote increased symptoms of orthorexia nervosa [14], a term coined to describe an obsession with proper or *healthful* eating [15]. Fitspiration content also promotes exercising for appearance-related reasons [16], which has been associated with negative body image [17], higher measures of disordered eating [18], and positive correlations with higher depressive and erectile dysfunction symptoms [19]. The aforementioned research often draws upon theoretical knowledge such as objectification [6,11] and social comparison theories [4,20] to explore the potentially negative implications of consuming fitspiration content on both mental and physical health.

The paradoxical findings on the relationship between fitspiration consumption and well-being reflect a small yet emerging body of research exploring the impact of fitspiration content on the lives and experiences of consumers. Despite this, research conducted on fitspiration to date focuses on the nature of the fitspiration content as opposed to the individuals that consume it [6,16,21]. While exploration into fitspiration content has valuable implications for policy, researchers have called for further inquiry exploring how individuals use fitspiration and the role it plays in their lives [2] to better support those who value it as meaningful content.

Currently, the majority of research exploring fitspiration focuses specifically on women’s experiences [11,22,23] and the nature of woman-centered content [4,24]. This is because women are more likely to use visual social media platforms [25,26] and are the biggest consumers of fitspiration images [21], and because fitspiration images are more likely to be women, with (36.4%) posts depicting female participants compared with 27.5% of posts depicting a male participant [21]. Associated findings demonstrate that exposure to fitspiration on Instagram may negatively influence women’s appearance-related concerns [11] and promote an often unrealistic and unachievable fit or thin body ideal [4,23,24]. These findings reflect broader cultural and media trends surrounding the ideal female body of one that is simultaneously very thin, and exceptionally fit and toned without displaying excess muscularity [16,27]. While fitspiration may be considered a healthy alternative to *thinspiration*, which communicates an unhealthy thin body ideal [24], the fitspiration ideal communicated to women within these contexts could be seen as oppressive, narrow, and contradictory and can promote women to engage in extreme exercise behavior [13]. There is also an emergent body of academic research that focuses on men’s experiences with fitspiration and the content that they are exposed to [21,28,29]. This research is valuable, as data suggest that a significant minority (30%) of fitspiration images on social media targets men [4,21]. Reviews of fitspiration content aimed at men reflect a heavily gendered muscular ideal [21,29] that provides a stark contrast to the message communicated to women through cultural discourse. Palmer [28] recognizes the pervasive nature of this form of social media content, demonstrating that it has the ability to influence men’s ideas regarding masculinity, and can encourage users to compare physiques to dominant *ideals.* This dominant discourse regarding male body ideology depicts participants who are highly muscular and nearly always have visible abdominal, bicep, and pectoral muscles, and compared with women these ideals demonstrate less emphasis on thinness or weight loss [21]. Therefore, the culturally pervasive ideological body identified in fitspiration targeting men reflects an ongoing cultural trend in the media, which has demonstrated a rapid increase in male muscle mass over time [30], aggregating the pressure on men to conform to increasingly unrealistic and unachievable body ideals.

Gendered analyses have also shown significant differences in the nature of the fitspiration images depicting men and women’s bodies, with women being more likely to be objectified and sexualized, and images with a greater focus on the participant’s buttocks [21], whereas posts of men were more likely to show the participant’s face [4]. Evidence also suggests that male fitspiration content on Instagram is more likely to focus on fitness as opposed to diet and alternative weight loss methods, with Tiggemann and Zaccardo [4] noting that 68.2% of male fitspiration focused on fitness-related activities, compared with only 58% for women. There are also clear gendered differences in how genders interact with and experience fitspiration content. In their study exploring male fitspiration use on Instagram, Fatt et al [29] identified that the frequency of viewing fitspiration posts was not significantly correlated with body satisfaction, appearance-based exercise motivation, and health-based exercise motivation in men. These results contrast with previous findings that demonstrated positive correlations between viewing frequency, body dissatisfaction, and disordered eating for women, signifying that fitspiration may psychologically impact men and women differently [11,13]. However, researchers have identified that fitspiration content consumption is linked to less body satisfaction when men internalize the muscular ideal and make more appearance comparisons [29]; therefore, certain men may experience the negative impacts of fitspiration in a similar way to women if they internalized male body ideals and made body comparisons.

### Objectives

The aim of this study is to explore how young (18-24 years) people engage with fitspiration content on Instagram and provide a gendered analysis of how and why they consume this content. This is in response to the aforementioned research that demonstrates the gendered nature of their consumption experiences in terms of the ideologies communicated through fitspiration, the composition of images, and the ways in which men and women interact with the content. While previous studies provide some light in terms of exploring how fitspiration consumption and the impact of fitspiration on individuals are gendered, because of the popularity and cultural relevance of this form of consumption, there is a great need for further research exploring the motivations for engagement, patterns of consumption, and perceived impact of fitspiration from a gendered perspective. This research will allow for greater exploration into how and why this experience is gendered, which can be used to help support digital literacy training and support for consumers of this content.

## Methods

### Study Design

The research used a cross-sectional web-based survey consisting of closed-ended questions to capture quantitative data to meet the study’s aim of exploring how those aged 18-24 years in the United Kingdom engage with fitspiration on Instagram using a gendered perspective. The survey was informed by the existing literature (outlined above), specifically by a previous survey-based fitspiration research study [2], and took approximately 10 minutes to complete. Ethical approval for the study was granted by an institutional ethical review (Bournemouth University).

### Setting

Data were collected during a 1-week period in May 2019 using a web-based insight exchange platform (Cint database) to request a representative sample from appropriate users registered to a range of web-based survey panels. A minimum sample size of 1000 was requested, which was achieved and exceeded, a benefit of using the insight exchange platform. However, it is important to note that the number of participants contacted is unknown owing to the nature of the participant recruitment platform.

### Participants

The eligibility criteria were that participants (n=1213) had to be aged 18-24 years (in line with previous research [21,31,32]) and broader demographic data [7,33], lived in the United Kingdom, and had reported viewing fitspiration on Instagram. In line with previous research, for the purposes of this survey, fitspiration was defined as photographs of fit people, people in the gym, health foods, or inspirational quotes relating to diet and fitness. Self-reported eligible participants also completed screening questions to ensure that they met the inclusion criteria (reporting a frequency other than *not at all* for the question “How often do you check Instagram?” or a frequency other than *never* to the question “How often do you view *fitspiration* on Instagram?”).

### Variables

#### Tested Variables

The following variables were measured (a copy of the questionnaire is available on request from the authors): demographic characteristics (age, BMI, gender, sexuality, and educational level), use (frequency of use and duration of use), and content and engagement (content engaged with while online, means of engaging with content, and reasons for engagement).

#### Demographic Characteristics

To allow for adequate description of the sample, a number of demographic characteristics were collected, including gender, age, sexual identity, and highest level of education completed. BMI was calculated using participants’ self-reported height and weight.

#### Instagram Use

To determine how the sample used Instagram, participants indicated how often they checked the platform using a 9-point scale adapted from previous research [11,17,29] (0: *not at all*; 9: every 5 minutes). In addition, participants were asked to specify how long they spent on Instagram on a typical day (*1-5 minutes or less* to *8 hours or more*) on an 8-point scale.

#### Fitspiration Use

To ascertain how the sample used fitspiration, participants indicated how often they viewed this content (as defined above) using scales adapted from previous literature [11] based on pilot study feedback on Instagram. The final scale had 8 points (1: never to 8: every time).

#### Fitspiration Content

Participants indicated the types of fitspiration they engaged with on the platform by selecting from a checkbox list informed by previous research [21] listing the following types of content: weight loss or fitness or body transformation journeys, personal trainers, athletes, celebrities or models, bodybuilders or strength content, clean eating, cleanses or detox, diet plans, fitness challenges, influencers, and fitspiration quote pages. Prevalent examples of fitspiration content were included, and participants were able to tick as many options that applied to them as it was anticipated (based on pilot study feedback) that they may have engaged with multiple forms of fitspiration.

#### Fitspiration Engagement

In line with previous fitspiration research [2] and based on feedback from the pilot study, participants identified how they engaged with fitspiration content by selecting from a list of nine engagement behaviors that ranged from active (post content; comment on posts; tag fitspiration accounts in posts; tag friends in posts; share content with friends; and like posts) to passive (follow accounts or view on Instagram feed, scroll through individual accounts posts or images, and search hashtags). Collecting these data helped address a research gap identified by Raggatt et al [2], who drew on the work of Jenkins-Guarnieri et al [34] to explain that previous studies investigating social media have not adequately captured the different ways in which people interact with social media content.

#### Reasons for Engaging With Fitspiration Content

Participants’ reasons for engaging with fitspiration content were collected using a predefined checkbox list of 8 reasons developed using existing literature [2] and based on feedback from piloting that included inspiration for health and well-being; healthy eating; to exercise to improve body shape, tone, or size; to gain muscle or get stronger; to diet or lose weight; to improve health and well-being; facilitate improvements in appearance; to help learn about health and well-being or because friends or peers view or like it.

#### Bias

A small incentive was offered in terms of points that participants could accumulate to gain future cash rewards, rather than simply offering cash incentives to try to minimize any subsequent bias. This is designed to discourage *professional* respondents whose primary motivation is to gain payment for completion.

### Analysis

Data were analyzed using IBM SPSS for Windows (version 25), with charts produced using Microsoft Excel. Given the nonparametric nature of the data, basic descriptive statistics were used to describe the nature of the sample, and chi-square analyses were undertaken to explore the relationships between gender and Instagram use in terms of the variables outlined earlier.

## Results

### Description of Sample

Following the screening questions, the survey was completed by 1213 eligible participants with a median age of 21 years. Response rates to individual questions varied and are reported as such. Where there were missing data, responses were deleted. A full breakdown is provided in Table 1; however, to summarize, most (68.10%) participants identified as female, compared with 28.19% identifying as male and 0.57% identifying as nonbinary or gender queer. In terms of sexuality, most of the sample identified as straight (83.47%). BMI calculations showed that over half of the respondents had a BMI within the normal range, 11.10% were categorized as underweight, and almost one-third (32.85%) were overweight or obese. Finally, most were educated to either Advanced or Advanced Subsidiary level (UK qualifications offered across a range of participants to school-leavers; 29.6%) or degree level or higher (33.70%).

**Table 1:** Description of sample.

Characteristics^a^		Values, n (%)
**Gender (n=1213)**		
	Female	826 (68.1)
	Male	342 (28.2)
	Nonbinary or genderqueer	7 (0.6)
**Sexuality (n=1090)**		
	Female straight	647 (59.4)
	Male straight	263 (24.1)
	Female bisexual or pansexual	106 (9.7)
	Male bisexual or pansexual	23 (2.1)
	Female gay	21 (1.9)
	Male gay	15 (1.4)
	Other	6 (0.6)
	Nonbinary and genderqueer, bisexual, and pansexual	4 (0.4)
	Nonbinary and genderqueer and gay	2 (0.2)
	Nonbinary and genderqueer and other	1 (0.1)
**BMI category (n=1108)**		
	Underweight	621 (56.0)
	Normal	232 (20.9)
	Overweight	132 (12.0)
	Obese	113 (11.1)
**Highest educational qualification (n=1184)**		
	Undergraduate degree or higher	399 (33.7)
	A or AS level	350 (25.3)
	GCSE^b^ or O level	207 (17.5)
	Higher qualification below degree level	181 (15.3)
	Postgraduate degree	113 (9.5)
	Other	47 (3.97)

^a^Responses are ordered from most to least common.

^b^GCSE: General Certificate of Secondary Education.

### Fitspiration Content

With regard to the fitspiration content on Instagram, 49.38% (599/1213) viewed content at least once per day, 32.32% (392/1213) at least once per week, and 18.30% (222/1213) less often. A total of 56.86% (714/1213) reported checking the platform at least once every hour, while 88.13% (1069/1213) checked it at least once every few hours. On average, 67.39% (812/1205) of participants spent an hour or more per day on Instagram with 18.50% (223/1205) spending over 4 hours and 2.99% (36/1205) spending over 8 hours on the platform. A categorical gender comparison between men and women (there were insufficient data to test differences with those who identified as nonbinary or genderqueer for any variables) using a chi-square test of independence demonstrated that women were more likely to check Instagram more regularly than their male counterparts (χ^2^_1, N=1168_=19.1; *P*=.008); however, there was no significant difference between the amount of time spent on Instagram per day for men and women (χ^2^_1, N=1160_=13.8; *P*=.06).

The most common types of fitspiration viewed by all participants were content from celebrities or models (717/1196, 59.95%), weight loss or fitness journeys or body transformation stories (641/1196, 53.60%), and influencers (473/1196, 39.55%). Gender differences in terms of engaging with specific forms of content are outlined in Table 2. Categorical gender comparisons between men and women using chi-square tests of independence showed male participants were more likely to view content posted by athletes (χ^2^_1, N=1153_=71.8; *P*<.001) and bodybuilders (χ^2^_1, N=1153_=32.82; *P*<.001) than their female counterparts and women were more likely to view content identified as weight loss or fitness journeys or body transformation stories (χ^2^_1, N=1153_=36.8; *P*<.001), celebrities or models (χ^2^_1, N=1153_=33.5; *P*<.001), clean eating (χ^2^_1, N=1153_=26.5; *P*<.001), cleanses or detox (χ^2^_1, N=1153_=30.8; *P*<.001), diet plans (χ^2^_1, N=1153_=11.9; *P*=.001), fitness challenges (χ^2^_1, N=1153_=6.9; *P*=.009) and from influencers (χ^2^_1, N=1153_=22.8; *P*<.001). The results are detailed in Table 2.

**Figure 1:**
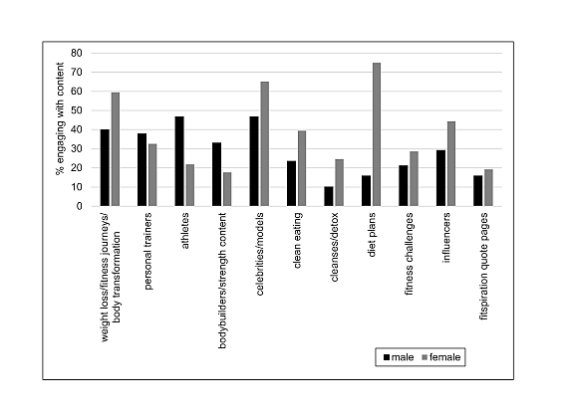
Gender and fitspiration content.

Figure 1 shows the gender and fitspiration content.

**Table 2:** Gender and fitspiration content (n=1153).

Type of engagement			Total (n=1153), n (%)		Gender, n (%)					*P* value				Φ coefficient	
					Male		Female								
**Engagement with weight loss or fitness journeys or body transformation content**											<.001				−0.179
	Do engage	620 (53.8)		134 (39.9)		486 (59.5)									
	Do not engage	533 (46.2)		202 (60.1)		331 (40.5)									
**Engagement with personal trainers**											.09				−0.050
	Do engage	393 (34.1)		127 (37.8)		266 (32.6)									
	Do not engage	760 (65.9)		209 (62.2)		551 (67.4)									
**Engagement with athletes**											<.001				−0.250
	Do engage	335 (29.1)		157 (46.7)		178 (21.8)									
	Do not engage	818 (70.9)		179 (53.3)		639 (78.2)									
**Engagement with bodybuilders or strength content**											<.001				0.169
	Do engage	255 (22.1)		111 (33)		144 (17.6)									
	Do not engage	898 (77.9)		225 (67)		673 (82.4)									
**Engagement with celebrities or models**											<.001				−0.170
	Do engage	689 (59.8)		157 (46.7)		532 (65.1)									
	Do not engage	464 (40.2)		179 (53.3)		285 (34.9)									
**Engagement with clean eating**											<.001				−0.152
	Do engage	401 (34.8)		79 (23.5)		322 (39.4)									
	Do not engage	752 (65.2)		257 (76.5)		495 (60.6)									
**Engagement with cleanse or detox**											<.001				−0.163
	Do engage	235 (20.4)		34 (10.1)		201 (24.6)									
	Do not engage	918 (79.6)		302 (89.9)		616 (75.4)									
**Engagement with diet plans**											.001				−0.102
	Do engage	258 (22.4)		53 (15.8)		612 (74.9)									
	Do not engage	895 (77.6)		283 (84.2)		205 (25.1)									
**Engagement with fitness challenges**											.009				−0.077
	Do engage	305 (26.5)		71 (21.1)		234 (28.6)									
	Do not engage	848 (73.5)		265 (78.9)		583 (71.4)									
**Engagement with influencers**											<.001				−0.141
	Do engage	460 (39.9)		98 (29.2)		362 (44.3)									
	Do not engage	693 (60.1)		238 (70.8)		455 (55.7)									
**Engagement with fitspiration quote pages**											.17				−0.041
	Do engage	210 (18.2)		53 (15.8)		157 (19.2)									
	Do not engage	943 (81.8)		283 (84.2)		660 (80.8)									

### How Participants Engaged With Fitspiration on Instagram

In terms of gender and engagement with fitspiration, a number of differences emerged, as highlighted in Table 3.

**Figure 2:**
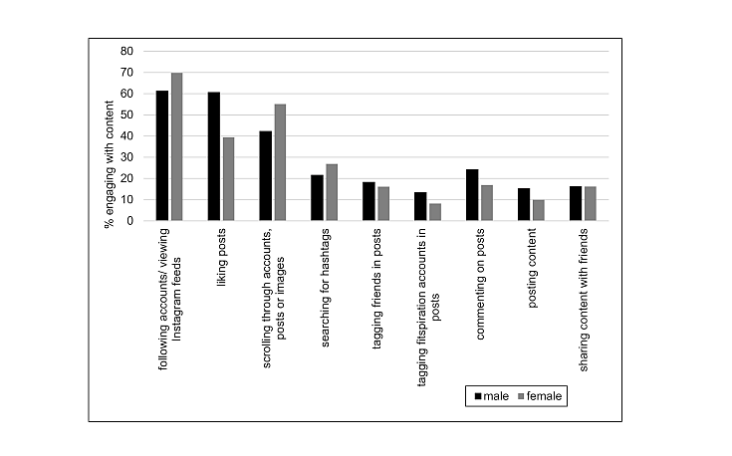
Gender and fitspiration engagement.

Figure 2 shows gender and fitspiration engagement.

Women were significantly more likely than men to engage in passive consumption behaviors such as follow accounts or views on Instagram feed (χ^2^_1, N=1139_=7.9; *P*=.005) or scroll through individual accounts, posts, or images (χ^2^_1, N=1139_=15.2; *P*<.001). Conversely, men were more likely than women to engage in active behaviors such as tag fitspiration accounts in posts (χ^2^_1, N=1139_=7.2; *P*=.007), comment on posts (χ^2^_1, N=1139_=8.1; *P*=.004), and post fitspiration content (χ^2^_1, N=1139_=6.4; *P*=.01).

**Table 3:** Gender and engagement with fitspiration (n=1139).

		Total (n=1139), n (%)	Gender, n (%)		*P* value		Φ coefficient	
			Male	Female				
**Engagement through following accounts or view on Instagram feed**						.005		−0.083
	Yes	767 (67.30)	202 (61.20)	565 (69.80)				
	No	372 (32.70)	128 (38.80)	244 (30.20)				
**Engagement through liking posts**						.11		−0.048
	Yes	731 (64.20)	200 (60.60)	531 (65.60)				
	No	408 (35.80)	130 (39.40)	278 (34.40)				
**Engagement by scrolling through individual accounts, posts, or images**						<.001		−0.115
	Yes	586 (51.40)	140 (42.20)	446 (55.10)				
	No	553 (48.60)	190 (57.60)	363 (44.90)				
**Engagement through searching for hashtags**						.06		−0.057
	Yes	289 (25.40)	71 (21.50)	218 (26.90)				
	No	850 (74.60)	259 (78.50)	591 (73.10)				
**Engagement through tagging friends in posts**						.39		0.026
	Yes	190 (16.70)	60 (18.20)	130 (16.10)				
	No	949 (83.30)	270 (81.80)	679 (83.90)				
**Engagement through tagging fitspiration accounts in posts**						.007		0.079
	Yes	110 (9.70)	44 (13.30)	66 (8.20)				
	No	1029 (90.30)	286 (86.70)	743 (91.80)				
**Engagement through commenting on posts**						.004		0.084
	Yes	217 (19.10)	80 (24.20)	137 (16.90)				
	No	922 (80.90)	250 (75.80)	672 (83.10)				
**Engagement through posting content**						.01		0.075
	Yes	130 (11.40)	50 (15.20)	80 (9.90)				
	No	1009 (88.60)	280 (84.80)	729 (90.10)				
**Engagement through sharing content with friends**						.96		0.002
	Yes	184 (16.20)	53 (16.10)	131 (16.20)				
	No	955 (83.80)	277 (83.90)	678 (83.80)				

### Why Participants Engaged With Fitspiration on Instagram

The main reasons for viewing fitspiration content selected by participants were to improve their health and well-being (669/1189, 56.27%); inspiration to exercise to improve body shape, tone, or size (636/1189, 53.49%); and inspiration for healthy eating (630/1189, 52.99%). There were clear gender differences between men and women in terms of the reasons for viewing fitspiration. Men were more likely to use fitspiration as an inspiration to exercise to gain muscle or get stronger (χ^2^_1, N=1147_=17.9; *P*<.001) or because friends or peers view or like it (χ^2^_1, N=1147_=5.5; *P*=.02) than women, and women were more likely to use fitspiration as inspiration for healthy eating (χ^2^_1, N=1147_=37.7; *P*<.001); inspiration to exercise to improve body shape, tone, or size (χ^2^_1, N=1147_=8.9; *P*<.001); or inspiration to exercise to diet or lose weight (χ^2^_1, N=1147_=13.5; *P*<.001) than their male counterparts. The reasons for engagement with fitspiration by gender are listed in Table 4.

**Figure 3:**
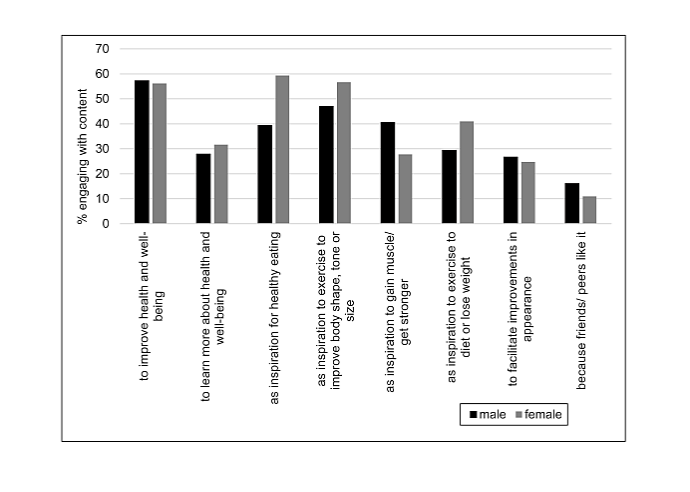
Gender and reasons for fitspiration engagement.

Figure 3 shows gender and reasons for fitspiration engagement.

**Table 4:** Gender and reasons for engagement (n=1147).

Reason for engagement			Total (n=1147), n (%)		Gender, n (%)				*P* value		Φ coefficient
					Male		Female				
**To improve health and well-being**									.74		0.010
	Yes	648 (56.50)		189 (57.30)		459 (56.20)					
	No	499 (43.50)		141 (42.70)		358 (43.80)					
**To learn more about health and well-being**									.20		−0.038
	Yes	351 (30.60)		92 (27.90)		259 (31.70)					
	No	796 (69.40)		238 (72.10)		558 (68.30)					
**As inspiration for healthy eating**									<.001		−0.181
	Yes	615 (53.60)		130 (39.40)		485 (59.40)					
	No	532 (46.40)		200 (60.60)		332 (40.60)					
**As inspiration to exercise to improve body shape, tone or size**									.003		−0.088
	Yes	618 (53.90)		155 (47)		463 (56.70)					
	No	529 (46.10)		175 (53)		354 (43.30)					
**As inspiration to exercise to gain muscle or get stronger**									<.001		0.125
	Yes	361 (31.50)		134 (40.60)		227 (27.80)					
	No	786 (68.50)		196 (59.40)		590 (72.20)					
**As inspiration to exercise to diet or lose weight**									<.001		−0.108
	Yes	432 (37.70)		97 (29.40)		335 (41)					
	No	715 (62.30)		233 (70.60)		482 (59)					
**To facilitate improvements in appearance**									.52		−0.019
	Yes	291 (25.40)		88 (26.70)		203 (24.80)					
	No	856 (74.60)		242 (73.30)		614 (75.20)					
**Because friends or peers like it**									.02		−0.069
	Yes	143 (12.50)		53 (16.10)		90 (11)					
	No	1004 (87.50)		277 (83.90)		727 (89)					

## Discussion

### Principal Findings

This quantitative survey of young Instagram fitspiration users reveals important insights regarding how and why consumers in the United Kingdom engage in this form of digital content. Most participants actively using Instagram for fitspiration (therefore eligible to take part in the study) were women (826/1175, 70.30%), consistent with previous academic research [21] and large-scale surveys [26], which suggests that women are more likely to engage with Instagram as a platform than men. However, this study makes a unique contribution to the existing body of knowledge in demonstrating that men and women have significantly different experiences in terms of what, how, and why they engage with the fitspiration content on Instagram. The findings relating to content preferences and motivations for viewing are gendered in a way that reflects clear dualistic gender ideals that are communicated through and reinforced by the digital content men and women consume, and the associated consumption behaviors.

### The Masculine Muscular Ideal

Historically, the dominant body ideal for men has increasingly shifted toward muscularity, with a specific emphasis on muscular definition and tone [35-37]. This trend has emerged as the masculine *muscular ideal*, which has been suggested to affect the ways in which many men view and critique their bodies [35]. In line with this, the study demonstrates that men are explicitly attracted to fitspiration content from athletes and bodybuilders, as inspiration to exercise to gain muscle and become stronger. These findings align with previous research that suggests web-based fitspiration images of men are far more likely to show bodies with visibly high levels of muscularity than those of women [21]. The study demonstrates that male fitspiration users are more likely to engage with content from professional sportsmen as opposed to models, celebrities, and influencers. This suggests that men may privilege fitspiration content from sports professionals with active performing bodies, as opposed to celebrities, models, and influencers who have perhaps constructed visually esthetic bodies to be admired and idealized.

Some scholarly work exploring the masculine body ideal has suggested that in addition to promoting muscularity, men are increasingly interested in reducing body fat to promote *leanness* [38]. Specifically, research has identified two separate dimensions of fat loss as a drive for thinness and a drive for leanness with men, perhaps having a tendency to strive toward a lean toned body as opposed to the thin ideal, which may be more attractive to women [39]. Despite research suggesting that men are increasingly interested in fat loss, the findings indicate that men are less likely to engage with content promoting weight loss and diet or use fitspiration to support healthy eating, diet, or weight loss than women. It could therefore be hypothesized that either fitspiration content promotes the *thin* as opposed to *lean* ideal or that diet or weight loss fitspiration content was predominantly focused on women and their bodies or lifestyles. Moreover, it could be suggested that fitspiration users are more likely to privilege a large muscular (as opposed to lean body) ideal compared with the general population. This aligns with previous research suggesting that male fitspiration users are significantly more likely to internalize a muscular body ideal when compared with nonusers [29]. While muscularity is still privileged in terms of male fitspiration, this could be down to this content focusing uniquely on achieving peak physical fitness, thus identifying a cultural prejudice toward traditional representations of masculinity [28].

### The Feminine Thin but Shapely Ideal

Critical research has suggested that the dominant body ideal for women is a contradictory body [4,23,24,27], simultaneously very thin, and exceptionally fit and toned without displaying excess muscularity [16]. This demonstrates a contrast to the masculine ideal body presented above, which honors muscular size. The notion that women privilege thinness is reinforced in this study, with female users being significantly more likely to consume fitspiration content promoting thinness through diet and weight loss than male users. This is also clear through women’s consumption motivation behavior, as they are significantly more likely to use fitspiration as inspiration to lose weight through diet and exercise than men. These preference and behavioral findings align with existing literature that demonstrates that the content of fitspiration aimed at women is more likely to show thin bodies and reflect a thin ideal than those aimed at men [4,21].

The findings also demonstrate that women are significantly more likely to consume fitspiration content from celebrities or models and influencers than their male counterparts, who, as noted above, are more likely to engage with athletes and bodybuilders. While numerous athletes and sportspeople monetize their use of Instagram [40,41], it could be argued that the promotion of a consumptive ethic and level of brand endorsement is more common within the accounts of influencers for whom this is often their sole intention or form of employment, and celebrities or models who increasingly yield salaries exclusively or in the main using social media. It could therefore be suggested that women who use fitspiration are likely to be marketed to or actively purchase goods to conform to an ideal body constructed in line with consumptive ethic, thus reinforcing the economic oppression that they have historically faced within Western society [42]. It could also be seen as reflective of the oppressive nature of the consumptive ethic [42] that emphasizes (active) male production and (passive) female consumption of goods and services, locating the power firmly in the hands of men.

### Gender and Active or Passive Engagement

While women are significantly more likely to engage in passive fitspiration consumption behaviors such as following, viewing, and scrolling through individual accounts, posts, or images, men are more likely to engage in active engagement, such as tagging fitspiration accounts in posts, commenting, and posting fitspiration content. This is in contrast to previous research [43], which suggests that women are consistently more likely to engage in social media activities by commenting, sharing, or liking. Therefore, based on these findings, it could be posited that fitspiration provides a unique social media context in which men engage more actively than women. This may be because of the emphasis on fitness and muscularity within fitspiration images reinforcing traditional masculine ideals, as opposed to content such as thinspiration, which promotes a thin ideal typically associated with femininity.

Ultimately, the finding that men are significantly more likely to report posting fitspiration content than women provides an interesting texture to the existing research narrative, as previous content research demonstrates that the majority (67%) of fitspiration images depict women as opposed to men [4]. There are a number of potential explanations for this. First, it may be that while men post more fitspiration content, it could be of images other than their own bodies, especially given that the fitspiration definition adopted is relatively broad (photographs of fit people, people in the gym, health foods, or inspirational quotes relating to diet and fitness). This could also be explained by drawing on research that explores the gendered nature of (self) objectification [11,39], which suggests that women are more likely to view their bodies as objects to be evaluated and may therefore be more inclined to offer images of their bodies for assessment. It may be that fewer women posted a higher volume of fitspiration images. This could be explained by the fact that women who meet gendered body ideals may be more likely to post a higher volume of images. Women not meeting gendered body ideals may be less inclined to label images of their bodies as fitspiration or failing to accurately report their active engagement or that men were posting images of women and coding them as fitspiration content.

### Study Strengths and Limitations

This study has numerous strengths and limitations that require consideration to adequately interpret the study findings. First, these findings reflect a large sample (n=1213) of fitspiration users in the United Kingdom and shed valuable light on their motivations for engagement, patterns of consumption, and the perceived impact of fitspiration from a gendered perspective. These user-centered data are especially enlightening, as they can be used to support the largely content-driven literature in this area. However, while this provides an alternative perspective, this study does not explain the relationship between content and consumption in terms of how they intersect, and to what extent consumption is driven by content. Furthermore, the research method provides a breadth of existing knowledge but sacrifices some depth that could potentially be achieved through qualitative interviews and questionnaires, or naturalistic observation and walk-through methods.

While the consumer-driven focus of the study is rationalized based on the absence of this focus from the existing research narrative, there are aspects of the consumer experience that have been neglected. More in-depth exploration could have involved exploring the gender differences in terms of body-part focus to build on existing literature [21] and by exploring the extent to which consumers engage with fitspiration images of bodies representing a gender other than their own; for example, women looking at images of men’s bodies. While there is a rationale for research in these areas, it was not considered to be the focus of the current research and therefore could be considered a limitation.

### Generalizability

As with any such survey, the representativeness of any sample can always be questioned. Although a random sample was drawn from a larger panel, it is difficult to judge the extent to which such samples are truly representative of the actual population; for example, it may be that women may be more likely to engage with web-based surveys; hence, the results must be treated with some caution. The use of nonvalidated measures could also be identified as a potential issue, even given the descriptive nature of the items, and this could be addressed in future research.

Third, this study explores the gendered nature of fitspiration consumption and yields some important findings regarding the differences in what fitspiration young UK-based men and women were consuming, and how and why they chose to consume it. However, because of the statistical nature of the analysis, this study did not go into any depth regarding individuals identified as nonbinary or genderqueer (7/1175, 0.6%) as opposed to males or females. This 0.6% is comparable with estimates for the general UK population that suggest 0.4% of adults in the United Kingdom identify as nonbinary or genderqueer when faced with it as an option alongside males and females [44]. Nonbinary or genderqueer individuals are likely to have unique experiences of consuming content largely related to the gendered bodies on the internet, and this was neglected in this study.

Finally, as with any study involving multiple analyses, the risk of false positives is always present. No adjustments were made for this within the analysis for two reasons: first, the research provides findings that are aligned with previous empirical work and theory, and second, that there was a general degree of consistency within the findings. While the risk remains, it would seem to be small, and given the exploratory nature of the study, further work and analysis may not be necessary to strengthen confidence in the analyses presented here.

### Future Directions

This study and the evaluation above highlight several valuable directions for future fitspiration research. First, there is a need for research to explore the relationship between content and consumption in terms of how they drive each other and the uniquely gendered nature of each. This could take the form of qualitative research exploring the fitspiration consumption process in more depth, or scholarly work focusing on fitspiration producers, who are likely to also be actively engaged consumers.

Furthermore, this survey could be used as a template to explore generational and cross-cultural differences and compare the unique ways in which these consumption patterns are gendered. It would be especially interesting to explore if and how this content is consumed in non-Western, majority world countries, with alternative ideas regarding body ideals and gender roles to make comparisons. In addition, to provide a comprehensive understanding of how representative this and other research on fitspiration use and UK consumers, there is a need for large-scale quantitative work that explores the prevalence of this web-based behavior both in the United Kingdom and globally.

While this research provides an exploration into the fitspiration consumption experiences of young people aged 18-24 years who represent around 24.1% of all UK-based Instagram users, additional research should focus on younger participants and adolescents who, despite using Instagram less (representing 8.1% of UK-based users [33]) may be more vulnerable to body ideals communicated via social media, and the negative aspects of fitspiration as they are at a crucial stage in the development of positive or negative body image [45].

There is also a need to explore how people who identify as nonbinary or genderqueer as opposed to male or female consume fitspiration, and the challenges that they face in being confronted by large volumes of this content that largely seems to be coded as either male or female. There is also a need for content-driven research exploring fitspiration content created by and for genderqueer and nonbinary individuals. It is also important that future research looks to further unpack the finding that women were more likely to consume fitspiration on Instagram, while men were more likely to create content. There is a need to explore possible explanations to ascertain whether men are less likely to post images of their bodies as fitspiration than women, such as posting images of women and coding them as fitspiration content that women fail to accurately report their active engagement, or suggest an alternative explanation. Finally, future research needs to explore whether male fitspiration users are indeed a unique subculture, and whether the male muscular ideal is broadly still dominant, as theorists have suggested that there is movement away from hypermasculine male stereotypes in the general media, with the strong hard man ideal becoming diminished in favor of more diverse forms of masculinity [46].

### Conclusions

The purpose of this study was to explore how young men and women engage with fitspiration content on Instagram and to provide a gendered analysis of how and why they consume this content. The key findings of this study achieved this aim in demonstrating the gendered way in which consumers engage with the fitspiration content on Instagram. Specifically, while all fitspiration consumers engaged with content that reinforced traditional body ideals, ideals were extremely gendered. Furthermore, male users were more likely to engage actively with content than female users who consumed content more passively, indicating that the experiences of men and women consuming fitspiration are vastly different. Therefore, based on these findings, it is suggested that any interventions designed to address the potential harm of this web-based content for young people should be gender-specific to adequately address the heavily gendered body ideals and experiential differences for young men and women.

## References

[1] (2019). Internet access households and individuals. Office for National Statistics.

[2] Raggatt M, Wright CJ, Carrotte E, Jenkinson R, Mulgrew K, Prichard I, Lim MS (2018). "I aspire to look and feel healthy like the posts convey": engagement with fitness inspiration on social media and perceptions of its influence on health and wellbeing. BMC Public Health.

[3] Vaterlaus JM, Patten EV, Roche C, Young JA (2015). #Gettinghealthy: the perceived influence of social media on young adult health behaviors. Comput Human Behav.

[4] Tiggemann M, Zaccardo M (2015). "Exercise to be fit, not skinny": the effect of fitspiration imagery on women's body image. Body Image.

[5] Easton S, Morton K, Tappy Z, Francis D, Dennison L (2018). Young people's experiences of viewing the fitspiration social media trend: qualitative study. J Med Internet Res.

[6] Tiggemann M, Zaccardo M (2018). 'Strong is the new skinny': a content analysis of #fitspiration images on Instagram. J Health Psychol.

[7] (2013). Number of monthly active Instagram users from January 2013 to June 2018. Statista.

[8] (2018). Technology tracker Q1 2018. Ipsos.

[9] #Fitspo. Instagram Explore.

[10] Manago AM, Graham MB, Greenfield PM, Salimkhan G (2008). Self-presentation and gender on MySpace. J Appl Dev Psychol.

[11] Fardouly J, Willburger BK, Vartanian LR (2017). Instagram use and young women’s body image concerns and self-objectification: testing mediational pathways. New Media Soc.

[12] Mayoh J (2019). Perfect pregnancy? Pregnant bodies, digital leisure and the presentation of self. Leisure Stud.

[13] Holland G, Tiggemann M (2017). "Strong beats skinny every time": disordered eating and compulsive exercise in women who post fitspiration on Instagram. Int J Eat Disord.

[14] Turner PG, Lefevre CE (2017). Instagram use is linked to increased symptoms of orthorexia nervosa. Eat Weight Disord.

[15] Orthorexia. National Eating Disorders Association.

[16] Boepple L, Ata RN, Rum R, Thompson JK (2016). Strong is the new skinny: a content analysis of fitspiration websites. Body Image.

[17] Vartanian LR, Wharton CM, Green EB (2012). Appearance vs. health motives for exercise and for weight loss. Psychol Sport Exerc.

[18] Adkins EC, Keel PK (2005). Does "excessive" or "compulsive" best describe exercise as a symptom of bulimia nervosa?. Int J Eat Disord.

[19] DiBartolo P, Lin L, Montoya S, Neal H, Shaffer C (2007). Are there healthy and unhealthy reasons for exercise? Examining individual differences in exercise motivations using the function of exercise scale. J Clin Sport Psychol.

[20] Peng C, Wu T, Chen Y, Atkin DJ (2019). Comparing and modeling via social media: the social influences of fitspiration on male Instagram users’ work out intention. Comput Human Behav.

[21] Carrotte ER, Prichard I, Lim MS (2017). "Fitspiration" on social media: a content analysis of gendered images. J Med Internet Res.

[22] Robinson L, Prichard I, Nikolaidis A, Drummond C, Drummond M, Tiggemann M (2017). Idealised media images: the effect of fitspiration imagery on body satisfaction and exercise behaviour. Body Image.

[23] Sumter SR, Cingel DP, Antonis D (2018). “To be able to change, you have to take risks #fitspo”: exploring correlates of fitspirational social media use among young women. Telemat Inform.

[24] Alberga AS, Withnell SJ, von Ranson KM (2018). Fitspiration and thinspiration: a comparison across three social networking sites. J Eat Disord.

[25] Duggan M, Ellison N, Lampe C, Lenhart A, Madden M (2015). Social media update 2014. Pew Research Center.

[26] (2021). Social media fact sheet. Pew Research Center.

[27] Markula P (1995). Firm but shapely, fit but sexy, strong but thin: the postmodern aerobicizing female bodies. Sociol Sport J.

[28] Palmer L (2015). Poppin bottles, getting wheysted:exploring young men’s engagement with fitspiration content and its consequential influences on attitudes and behaviour. J Promotional Commun.

[29] Fatt SJ, Fardouly J, Rapee RM (2019). #malefitspo: links between viewing fitspiration posts, muscular-ideal internalisation, appearance comparisons, body satisfaction, and exercise motivation in men. New Media Soc.

[30] Law C, Labre MP (2002). Cultural standards of attractiveness: a thirty-year look at changes in male images in magazines. Journal Mass Commun Q.

[31] Bell BT, Cassarly JA, Dunbar L (2018). Selfie-objectification: self-objectification and positive feedback ("Likes") are associated with frequency of posting sexually objectifying self-images on social media. Body Image.

[32] Jong ST, Drummond MJ (2016). Exploring online fitness culture and young females. Leisure Stud.

[33] UK: Instagram users by age. Statista.

[34] Jenkins-Guarnieri MA, Wright SL, Johnson B (2013). Development and validation of a social media use integration scale. Psychol Pop Media Cult.

[35] McCreary D (2002). Gender and age differences in the relationship between body mass index and perceived weight: exploring the paradox. Int J Men's Health.

[36] Darcy AM, Doyle AC, Lock J, Peebles R, Doyle P, Le Grange D (2012). The eating disorders examination in adolescent males with anorexia nervosa: how does it compare to adolescent females?. Int J Eat Disord.

[37] Mellor D, Waterhouse M, Mamat NH, Xu X, Cochrane J, McCabe M, Ricciardelli L (2013). Which body features are associated with female adolescents' body dissatisfaction? A cross-cultural study in Australia, China and Malaysia. Body Image.

[38] CD M, GJ G (2017). Body image concerns of male rugby players, with specific focus on muscularity and body fat. J Obes Overweig.

[39] Smolak L, Murnen SK (2008). Drive for leanness: assessment and relationship to gender, gender role and objectification. Body Image.

[40] Smith LR, Sanderson J (2015). I'm Going to Instagram It! An analysis of athlete self-presentation on Instagram. J Broadcast Electron Media.

[41] Toffoletti K, Thorpe H (2018). The athletic labour of femininity: the branding and consumption of global celebrity sportswomen on Instagram. J Consum Cult.

[42] Wolf N (2013). The Beauty Myth.

[43] Blank G, Lutz C (2016). The social structuration of six major social media platforms in the United Kingdom: Facebook, LinkedIn, Twitter, Instagram, Google+ and Pinterest. Proceedings of the 7th 2016 International Conference on Social Media.

[44] Glen F, Hurrell K (2012). Technical Note: Measuring Gender Identity. Manchester: Equality and Human Rights Commission.

[45] Voelker DK, Reel JJ, Greenleaf C (2015). Weight status and body image perceptions in adolescents: current perspectives. Adolesc Health Med Ther.

[46] Hanke R (1998). The “mock‐macho” situation comedy: hegemonic masculinity and its reiteration. West J Commun.

